# Assessing team mindsets: Development and validation of a scale for team learning

**DOI:** 10.1186/s40359-025-03906-3

**Published:** 2026-01-16

**Authors:** Soo Jeoung Han, Mirim Kim

**Affiliations:** 1 Graduate School of Education, Yonsei University, Seoul, South Korea; 2 Department of Psychology, Sungshin Women’s University, Seoul, South Korea; 3 Human-Centered AI Institute, Sungshin Women’s University, Seoul, South Korea

**Keywords:** Team mindset scale development, Fixed and growth mindset, Team learning

## Abstract

Team mindsets are the collective beliefs of team members regarding their ability to enhance each other’s capacity by sharing knowledge, learning from failures, and collaboratively addressing challenges. This study developed and validated the Team Mindset Scale (TMS) to measure individuals’ perceptions of teamwork in relation to learning. The initial study revealed that the developed 48-team mindset items represented a two-factor structure encompassing team growth and fixed mindsets. We conducted an exploratory factor analysis on government survey data from 91 employees and identified two factors representing team mindsets. We verified the factor structure again and examined the psychometric properties of the items using a larger sample of 908 college students who participated in team projects. In the second study, we tested the two-factor structure of team mindsets and the participants’ response biases. In the third study, we conducted confirmatory factor analysis models to cross-validate the two-factor model and investigated measurement invariance across genders. Furthermore, we assessed the convergent and discriminant validity of team mindset constructs by evaluating the relationship between Dweck’s individual mindsets and the newly identified team mindsets. The scale we developed can enhance future research on team mindsets and offer valuable insights for practitioners and educators.

## Introduction

Researchers have extensively examined mindset in the contexts of learning and education [1–3]. For example, Dweck and Leggett [4] introduced the concept of mindset as a unidimensional continuum between fixed and growth mindsets, based on implicit person theory, which aids in comprehending the nature of individuals’ intelligence, values, and talents. Comprehending the nature of an individual’s mindset is imperative for both individual and organizational success [5, 6]. The fundamental premise of a growth mindset is that individuals possess the potential to develop their intellect and increase their talent. Conversely, a fixed mindset posits that one’s abilities and intelligence are fixed or even inherent [7].

Individual growth mindsets represent a perspective that perceives challenges as opportunities rather than tests of inherent abilities; it also emphasizes the effort invested in learning and learns from failures [8, 9]. An emerging and effective approach to engender positive results is to experiment, learn from mistakes, and collaboratively seek innovative solutions, in accordance with the pursuit of a growth mindset [7]. Consequently, researchers have endeavored to broaden mindset theory in learning to include individual creativity [10] and to ascertain methods for revealing mindsets within larger teams [11], alongside other conceptualizations of mindsets. Such adaptation of the individual growth mindset concept to team mindsets is a novel approach. However, the literature lacks an examination of team mindsets.

First, although researchers have extensively examined the concept of mindsets at the individual level, the existing literature inadequately depicts individuals’ perceptions of team mindsets [12]. As previous studies of team mindsets evolved [12–14], researchers defined team mindsets as the collective beliefs of team members regarding their ability to enhance each other’s capacity by sharing knowledge, learning from failure, and collaboratively surmounting challenges [13, 14]. Team mindsets imply that mindsets can influence individuals’ self-perceptions as well as their judgments of others [15]. Further investigation into the definition and development of metrics for team mindsets can enhance researchers’ comprehension of mindsets across various levels.

Second, researchers have extensively investigated the impact of a growth mindset within K–12 educational contexts; however, this exploration is comparatively limited in contexts that promote group learning, such as higher education [15–18] or workplace learning [11]. Despite the significance of teams in modern organizations and the role of higher education in equipping students for teamwork, team-level characteristics based on adult group behavior, such as team mindset for team function or performance, remain under-studied. However, several pioneering studies have indicated potential correlations between individual and team mindsets [11, 19, 20]. In other words, individual beliefs about learning may shape how they perceive team members’ capacity to grow and collaborate, creating a link between individual and team-level mindset development. Given that higher education increasingly relies on team-based learning to develop students’ collaboration, problem-solving, and communication skills for future professional environments, it is important to investigate how individual and team mindsets are connected and to identify the core dimensions of team mindsets.

To address this gap, we developed the Team Mindset Scale (TMS) to measure individuals’ perceptions of teamwork and team dynamics among two adult cohorts—workplace employees and higher-education students—because both contexts rely on continuous learning and collaboration, yet existing research has treated them separately. Analyzing these cohorts together can enable us to test whether the construct demonstrates similar meaning across contexts and addresses the gap in adult-based mindset research beyond K–12 settings. Therefore, this study aimed to answer the following research questions: (1) How do team mindsets differ from individual mindsets in terms of their factor structures? (2) What are the key dimensions of team mindsets, and how can they be measured reliably? (3) Does the TMS demonstrate robust psychometric properties, including construct validity and measurement invariance?

To answer these questions, we conducted three sequential studies to examine the psychometric properties of the TMS and sought to enhance team mindsets in future group studies. Study 1 focused on conceptual differentiation, identifying that team and individual mindsets exhibit distinct factor structures, supporting the need for a team-specific mindset scale. Study 2 examined item-level psychometric properties to refine the TMS, ensuring its validity and reliability. Study 3 further tested the validity of the TMS by assessing measurement invariance, as well as convergent and discriminant validity, confirming that team mindsets function as a distinct psychological construct.

Our findings contribute to the theoretical foundation of team mindset research by clarifying its conceptual boundaries and developing a validated measurement tool. By bridging the gap between theory and application, the TMS provides a practical instrument for educators, administrators, and researchers to assess and cultivate effective team mindsets in educational and organizational settings. The TMS enhances comprehension of the impact of team mindsets on learning and offers practical applications for optimizing team-based projects and institutional outcomes in education.

## Conceptual framework

### Mindsets

Mindset theory from the early research of Dweck [7] and associates, aimed at comprehending the behavioral patterns linked to motivation. The conceptual underpinning of this study is the implicit theory, which comprises entity implicit and incremental implicit theories. Individuals’ convictions regarding the malleability of their attributes, such as intelligence or personality, as well as personal characteristics (e.g., intelligence, various forms of ability, and personality), form the basis of implicit theories [21]. For instance, a prototypical *entity* implicit theory—recently termed a fixed mindset [7]—posits that such personal attributes constitute a largely stable entity that typically exhibits minimal change over time [22]. In contrast, prototypical *incremental* implicit theorists, commonly known as individuals with a growth mindset [7], generally hold the belief that individuals can evolve and develop their behavior over time, especially when they commit to learning and implementing more effective strategies for task execution [22]. Apart from these definitions, Dweck [7] and others have further developed and refined instruments for measuring people’s attributional perspectives on ability, creating a scale that identifies a spectrum ranging from a fixed mindset to a growth mindset.

Most empirical studies employ a mindset definition akin to Dweck’s original description [2, 11, 22, 23]. Dweck’s [9] fixed mindset (or entity theory) posits that individuals perceive human attributes as fixed traits; hence, they accept their abilities as static. For instance, an individual may believe that a person may believe that they are inherently endowed with certain behaviors, personalities, or character traits, and that they possess no capacity to modify them. Fixed-mindset students prioritize outcomes and screen out negative feedback. In comparison, individuals with a growth mindset (or incremental theory) assert that anyone may substantially enhance their through effort and education, and that all people can evolve in terms of their behavior, personality, or character over time [24]. Students with a growth mindset concentrate on the process, actively seeking and responding to feedback [7]. The growth and fixed mindsets can be positioned on a continuum, with the fixed mindset end denoting immutable and stable personal attributes, and the growth mindset end signifying the capacity for development and enhancement through effort and learning.

### Team mindsets

Despite the robustness of individual mindset theory, relatively limited theoretical work has been conducted on team mindsets because the construct has only recently emerged in the literature and has lacked a validated instrument for empirical testing. However, team mindsets are theoretically significant because they shape how members collectively interpret challenges, support one another’s learning, and pursue improvement as a group—processes that are central to effective teamwork in both educational and organizational settings. Team mindset can be conceptualized as the *shared beliefs among team members about the team’s capacity to develop and improve through effort and learning* [12–14]. While individual mindset focuses on personal growth and traits, team mindset refers to how individuals perceive their team’s collective orientation toward learning, feedback, and adaptability.

Recent qualitative research by Han et al. [13, 14] proposed a conceptual framework for team mindsets, distinguishing them from similar constructs like team efficacy, psychological safety, or shared mental models. These studies identified eleven dimensions of team mindset through focus group interviews and thematic analysis: (1) receptiveness to feedback, (2) eagerness to learn, (3) learning from mistakes, (4) effort, (5) openness to change, (6) persistence, (7) resilience, (8) embracing challenges, (9) mutual trust, (10) acceptance of others, and (11) contribution to others’ welfare. These dimensions reflect how teams collectively interpret challenges, feedback, and development opportunities—not merely how individuals behave.

Although team mindset is related to individual mindset, it is not a simple aggregation of individuals’ beliefs. Rather, it captures shared cognitive frames within a team. For example, Gutshall [19] found that trust among team members can moderate how individual beliefs affect team learning, and Dweck [22] emphasized how a leader’s mindset influences team dynamics and performance evaluations. Thus, team mindset is both conceptually and functionally distinct from its individual-level counterpart.

We posit that a team growth mindset may enhance team learning behaviors. Researchers examined these behaviors as a proficient team process that enables teams to adapt to evolving circumstances while enhancing knowledge, products, and services [25]. Edmondson [26] characterized team learning behavior as “an ongoing process of reflection and action marked by asking questions, seeking feedback, experimenting, reflecting on results, and discussing errors or unexpected outcomes of actions” (p. 353). However, a disparity exists between individual and team behaviors that influence overall team efficacy. Examining a team mindset reveals the connection between individual mindsets, team mindsets, and resultant behaviors. Team-level behaviors do not occur spontaneously; thus, understanding the emergence of these behaviors from individual and team mindsets is essential for enhancing team effectiveness as a cohesive unit.

To date, there has been no validated instrument explicitly designed to measure team mindset as a multidimensional, team-level construct. Some studies have explored the influence of leaders’ or managers’ growth mindsets on subordinates’ outcomes, such as organizational citizenship behaviors [11, 27, 28] but they do not directly measure team mindset. Therefore, this study addresses that gap by developing and validating the Team Mindset Scale (TMS), which measures individuals’ perceptions of their team’s mindset—how “we as a team” approach challenges, learning, and improvement. The scale includes both Team Growth Mindset (TGM) and Team Fixed Mindset (TFM) dimensions. The item pool was derived from the eleven conceptual dimensions proposed in prior qualitative studies [13, 14], and items were refined through expert review for content validity. These eleven dimensions represented diverse behavioral expressions of team mindsets; however, during scale development, exploratory and confirmatory factor analyses showed that the items converged into two higher-order dimensions—Team Growth Mindset and Team Fixed Mindset—reflecting the underlying latent structure of the construct. Responses are collected using a five-point Likert scale, and items are phrased to capture perceived shared beliefs and behaviors at the team level.

This paper presents the results of a multi-phase validation process. In Study 1, we conducted exploratory factor analysis to determine the scale’s structure and examined how team mindsets differ from individual mindsets. Studies 2 and 3 tested the psychometric properties of the refined instrument using new samples, assessing item-level reliability, measurement invariance, and construct validity. In doing so, this research contributes to theory-building in team learning and performance by offering a theoretically grounded, empirically validated framework for understanding and measuring team mindset.

## The present studies

We executed three studies to create a scale for measuring team mindsets and to establish (a) construct validity based on its unique factor structure (Study 1), (b) cross-validation using a definitive set of items with satisfactory item characteristics (Study 2), and (c) to demonstrate further construct validity of the final scale by testing measurement invariance and the factor structure alongside other constructs (Study 3). We conducted our analyses using M*plus* 8.5 [29].

### Study 1: Identifying team mindsets

Compared to individual mindsets, team mindsets are relatively new constructs. Han et al. [13] and Han, Jin, and Oh [14] proposed that team mindsets encompass sub-dimensions, asserting that these mindsets transcend the mere aggregation of individual mindsets at the team level. Study 1 elucidates the divergent perceptions of participants regarding individual and team mindsets. We used exploratory factor analysis (EFA) to examine potential team mindset constructs and distinguished them from individual mindset constructs.

### Methods

We recruited 400 employees from a traffic division in the United States. They belonged to the same public works department; therefore, we presumed analogous team characteristics. Among them, 101 employees engaged in the study. Due to the incompleteness of certain responses to target measures, we utilized 91 complete responses for the analysis. Most participants were male (62%) and white (47.8%).

### Measures

#### Team mindset

We initially developed 48 items derived from six sub-dimensions that constitute team mindsets based on the previous studies [13, 14]. Individual mindset items asked respondents to reflect on their own personal beliefs, using first-person singular pronouns (e.g., “I”). In contrast, team mindset items used first-person plural pronouns (e.g., “our”) to assess the respondent’s perception of their team’s collective beliefs and attitudes. Thus, the team mindset construct was measured at the individual level but reflected an individual’s perception of “our mindset as a team.” Each 24-item set exemplified a team growth mindset (TGM) and a team fixed mindset (TFM) within the sub-dimensions. The number of items for each dimension were as follows: perseverance (PERS, 4), openness to change (CHG, 4), risk-taking (RISK, 3), receptiveness to feedback (FDB, 4), learning from mistakes (MIST, 4), and eagerness to learn from the team (DESR, 5). For example, we designated the second item under the openness to change for each team growth and fixed mindset as TGCHG2 and TFCHG2 (refer to Table 7). We scored all team mindset items on a five-point Likert scale (ranging from 1 = *strongly disagree*, 5 = *strongly agree*).

#### Individual mindset

This study focused on team mindsets, although the initial concept of the mindset scale originated at the individual level [24]. Moreover, scholars have extensively examined the psychometric attributes of the individual mindset scale [7]. Utilizing a five-point Likert scale, we measured growth and fixed mindsets for each of the four items. In Study 1, Cronbach’s alpha for the growth mindset was 0.95, while it was 0.93 for the fixed mindset.

### Procedure

We explored the potential number of factors based on parallel analysis and a scree plot. The parallel analysis is to find a meaningful pattern derived from the factors by comparing the eigenvalues between research dataset (i.e., sample) and the random dataset. If a certain number of factors explain the developed scale, the eigenvalue pattern needs to be different from the monotonous pattern from the random dataset, and the scree plot is a tool showing such patterns graphically. Following this rationale, we decided *i*-factor model if the eigenvalue of the sample was smaller than the random eigenvalue at (*i +* 1)^th^ factor [30].

First, we conducted EFA exclusively on team mindset items to ascertain the quantity of team mindset factors. Subsequently, we incorporated individual mindset items into the factor analysis to examine how the number of factors varied with two different types of mindsets. This process was to identify how the number of factors varied if team mindset and individual mindset items were analyzed together, and to examine the discriminant validity between the team mindsets and the individual mindsets. We performed an EFA utilizing oblique goemin rotation [31], the default option in M*plus*.

### Results

**Fig. 1:**
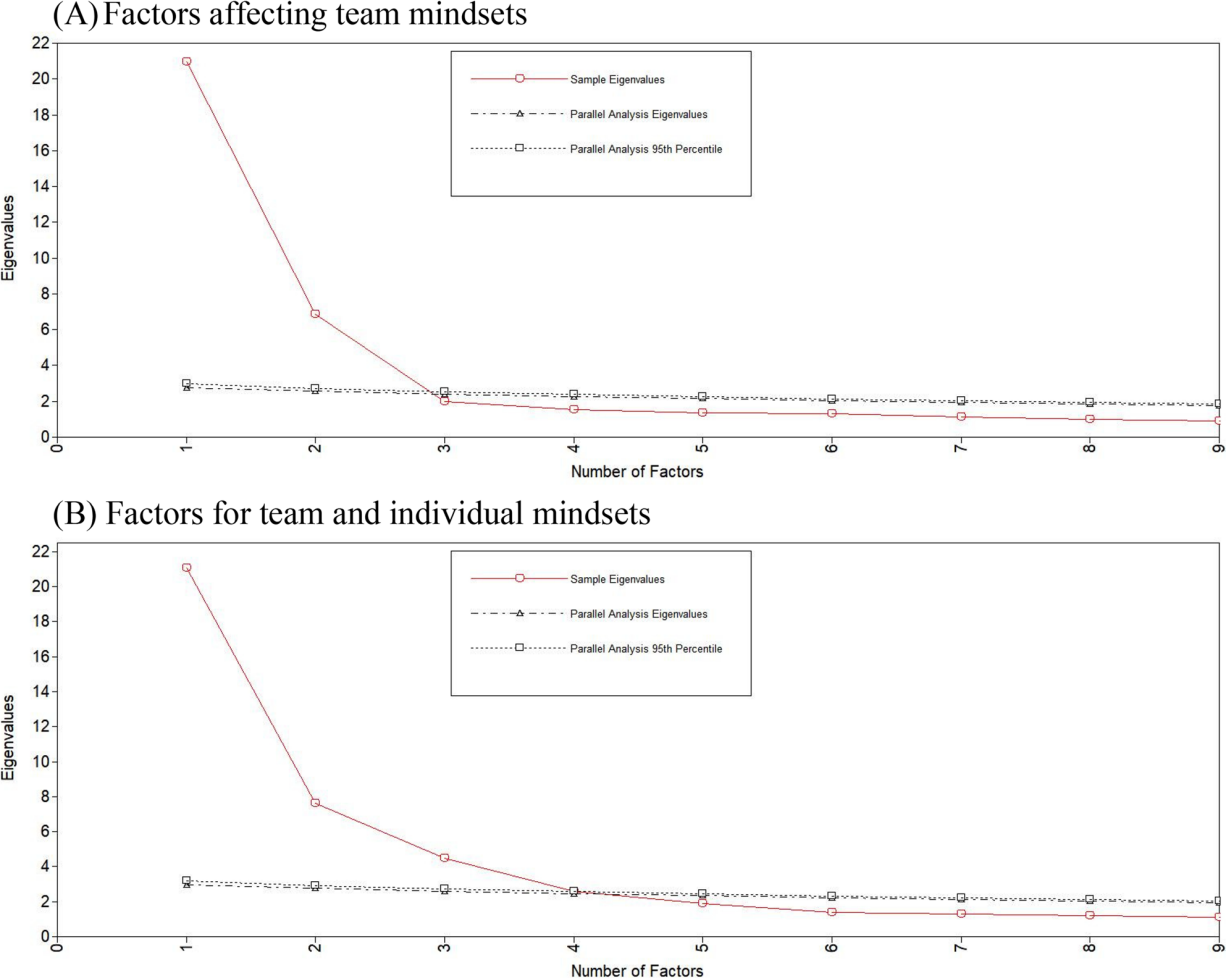
Scree Plots and Parallel Analysis

Figure 1 illustrates the scree plots and parallel analyses of the EFA. Upon analyzing 48 items pertaining to team mindsets, the scree plot (A) exhibited pronounced declines following the initial two factors, with the slope of the plot progressively diminishing. The eigenvalue derived from the parallel analysis exceeded the actual sample eigenvalue for the third factor; therefore, two factors were feasible for the team mindsets. The following scree plot (B) illustrated the difference resulting from the inclusion of eight individual mindset items in the analysis. The parallel analysis indicated a higher eigenvalue than the sample eigenvalue at the fifth-factor juncture. This outcome indicates four factors influencing both team and individual mindsets. The number of factors may indicate that individuals within the team workplace perceived team and individual mindsets dissimilarly.

Table 1 presents the factor loadings derived from the four-factor structure, specifically detailing the factor loadings for the individual mindset items. The final factor structure of team mindsets will be reported in future studies after testing the psychometric properties of the recent set of items. Each four-item group of individual growth and fixed mindset exhibited substantial factor loadings on Factor 1 and Factor 2. Therefore, we anticipated that the corresponding factors would be individual growth and fixed mindsets. However, the items exhibited no substantial factor loading on the subsequent two factors (i.e., Factor 3 and Factor 4). This result implied a minor correlation between individual mindset items and team mindset items, revealing distinct factor structures.

**Table 1:** Part of factor loadings for individual mindsets

	Factor 1		Factor 2		Factor 3		Factor 4	
	Estimates	*p*-value	Estimates	*p*-value	Estimates	*p*-value	Estimates	*p*-value
INGM1	**0.893**	< 0.001	0.018	0.750	0.002	0.797	−0.072	0.314
INGM2	**0.953**	< 0.001	−0.036	0.630	0.048	0.322	0.006	0.785
INGM3	**0.937**	< 0.001	−0.059	0.457	0.014	0.736	−0.005	0.789
INGM4	**0.863**	< 0.001	0.007	0.790	0.015	0.760	0.077	0.342
INFM1	−0.323	0.087	**0.775**	< 0.001	0.008	0.788	0.026	0.708
INFM2	**−0.410**	0.025	**0.829**	< 0.001	−0.007	0.781	0.000	0.798
INFM3	−0.313	0.112	**0.809**	< 0.001	−0.010	0.780	−0.007	0.790
INFM4	−0.327	0.082	**0.770**	< 0.001	0.037	0.613	0.065	0.439

*INGM *= Individual growth mindset; *INFM* = Individual fixed mindset; Bold indicates statistically significant point estimates

## Study 2: Screening items of team mindsets

Study 2 verified the two-factor structure of team mindsets using another sample and clarified items based on psychometric properties to develop a TMS with the validated item sets. We examined item characteristics based on EFA to investigate the item-factor relationship and employed an item response tree (IRTree) [32] model to identify response biases in the items. Furthermore, we evaluated content validity, which reflects the adequacy of the content domain of items, to determine the optimal item set for the Team Mindset Scale.

### Methods

#### Participants

The participants comprised undergraduate and graduate students from a public research university in the United States. We recruited them via random emails in the spring of 2019. The target participants had participated in class team projects within the preceding six months. The email requested students to provide their demographic data and their views on team and individual mindsets during team activities. Out of 1,297 responses, we utilized 908 for analysis after excluding incomplete responses that lacked data on the target scale. We randomly divided the data into 40% and 60%, which were utilized for Studies 2 and 3, respectively. Study 2 utilized 40% (*N* = 363, Female = 66%, White = 82%) of the entire data to repeat the EFA in Study 1 and amend the item sets. Study 2 re-evaluated the number of factors and examined the characteristics of the items. Conversely, Study 3 validated the refined item set through sequential structural equation modeling; thus, we allocated additional data to Study 3.

### Measures

We employed the same 48 team mindset items as utilized in Study 1. Without excluding any items, Cronbach’s alphas based on the 24-item TGM and TFM were 0.94 and 0.78, respectively.

### Procedure

Study 2 replicated the EFA using identical procedures and standards (i.e., scree plot and parallel analysis) as employed in Study 1. We meticulously examined factor loadings to assess each item for the factors. Additionally, we re-examined content validity to identify possible misfits of the factor analysis model based on the item contents. We assessed additional item properties by examining two response biases (i.e., midpoint and extreme response styles) using the IRTree model. We screened out certain items from the TMS based on the results and did not include them in Study 3.

### Results

#### Exploratory factor analysis

The preliminary EFA findings suggested a three-factor model for team mindsets. On the one hand, all TGM items loaded on the first factor were statistically significant, demonstrating that the TGM factor accounted for the 24-item TGM. On the other hand, TFM items were loaded onto two factors, indicating that the developed items of TFM did not associate well under a single TFM construct. This result may indicate two possibilities: (a) the existence of two sub-dimensions for TFM, or (b) certain items were unrelated to TFM. In the latter scenario, an additional tentative factor accounted for the residual variance of the items not loaded on the TFM factor. To investigate the source of the misfit, we examined the factor loading size and its direction based on the EFA results and re-evaluated the item contents.

During the EFA phase, we deemed one item for the TFM (i.e., TFPERS3) unpromising due to its factor loading being below 0.3 across all factors; the remaining items exhibited no concerns regarding factor loading size. We based the team and individual mindsets on a common framework in which both growth and fixed mindsets exist along a single mindset continuum. In other words, we assumed that growth and fixed mindsets to be closely connected, yet fundamentally opposed and moving in opposite directions. Upon evaluating this framework, we determined that the four-item TGM (i.e., TGCHG3, TGRISK3, TGFDB2, and TGMIST3) and the eight-item TFM (i.e., TFPERS2, TFCHG1, TFCHG3, TFRISK2, TFRISK3, TFFDB2, TFDESR1, and TFDESR2) were unsuitable due to their uniform direction of factor loadings across factors. Despite the inadequacy of the 13 items from TGM and TFM, we re-evaluated the other item properties in Study 2; however, we excluded them from the analysis in Study 3.

#### Content validity

The American Educational Research Association [33] defines validity as “the degree to which evidence and theory support the interpretation of test scores for proposed uses of tests” (p. 11) and refers to various forms of validity evidence. The present study EFA to assess the internal structure of the TMS for the purpose of testing construct validity. Moreover, we assessed content validity to examine the relationship between the test content and the constructs of team mindsets. This study evaluated content validity as qualitative validity evidence [34], focusing on the extent to which the TMS accurately represented the domain of team mindsets on the scale.

First, we considered face validity, which reflects the degree to which the items appeared valid and adequate to the test participant [34]. The respondents indicated that three items lacked sufficient clarity to enable them comprehend the question’s intent (TGDESR4, TGDESR5, and TFDESR4). Second, the content-related experts reviewed the item contents, including themes, wording, or format [33], and decided to eliminate certain items. It is important to acknowledge that we initially developed the items based on the mindset continuum; we formulated the TGM and TFM items to inquire about the various extremes of the continuum. For example, TGFDB4 and TFFDB4 constituted the fourth item pair within the “accepting feedback” sub-dimension, inquiring whether the respondent’s team was receptive to diverse arguments. TGFDB4 directly inquired about “multiple arguments,” whereas TFFDB4 included the passage of “dominant members’ opinions.” The content experts concluded that items were deemed inappropriate if the paired items contained precisely opposing statements or wordings because their contradictory nature would result in redundancy. In these instances, we chose one of the two items that conveyed a more explicit meaning or formulated a neutral statement devoid of significantly positive or negative language to obviate social desirability bias among respondents. Consequently, we eliminated three items (TGCHG4, TFFDB3, and TFMIST2) in accordance with this criterion. Furthermore, we excluded TGPERS4, which asked about limited resources, as it may not be applicable to all respondents’ circumstances.

#### Item response tree model

We found that most participants’ responses fell in the 4–5 categories (i.e., *agree* and *strongly agree*), signifying robust concurrence with the statement across items. The ideal scenario is that a strong consensus accurately reflects the elevated levels of TGM and TFM. However, response biases may have influenced the high-agreement pattern, as respondents tended to overestimate or underestimate the observed scores due to a preference for a specific category, irrespective of their latent trait level. This approach may compromise the measurement of target latent traits [35, 36] and result in diminished reliability and validity [37, 38]. In Study 2, we employed the IRTree model to identify potential response biases and examine their effects on agreement patterns. We investigated the tendency to select the midpoint (i.e., *neither agree nor disagree*) or extreme categories (i.e., *strongly (dis)agree*) irrespective of team mindsets to check response bias.

**Fig. 2:**
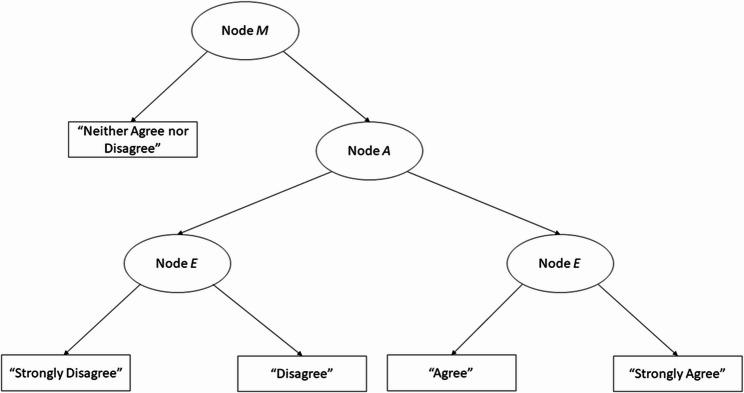
Tree Diagram of Three Nodes for a 5-Point Likert Scale. *Node M =* Midpoint response style; *Node A* = Agreement with the item content; *Node E* = Extreme response style

The IRTree model reflects a sequential decision-making process concerning ordinal categories organized in a tree structure. The process for each category persists until a branch of the sub-tree reaches the final node/query, which corresponds to the observed value for that category [39, 40]. Figure 2 illustrates the three-node model for the five-point Likert scale utilized in Study 2. Böckenholt [41] asserts that an odd number of categories increases the likelihood of midpoint response tendencies compared to an even number of categories. We adopted this theoretical framework [39, 42] and assumed that participants initially selected the midpoint category (i.e., Node *M*). If their response did not correspond to the midpoint value, the process progressed to the adjacent categories on the left or right via Node *A* to indicate their (dis)agreement, with their ultimate decision articulated as *(dis)agree* or *strongly* (dis*)agree* through Node *E*.

The general item response theory (IRT) model applicable to the Likert scale is a graded response model (GRM) [43]. Therefore, we evaluated and contrasted the GRM and IRTree models to ascertain which model more accurately represented the item properties and whether the hypothesized response biases affected the items. We established the GRM to embody two team mindsets and employed the IRTree model to illustrate the two team mindsets and two response biases. In the IRTree model, we used the one-parameter IRT model for the two response biases and the two-parameter IRT model for the two team mindsets. The one-parameter model assessed the item’s difficulty, whereas the two-parameter model estimated each item’s difficulty and discrimination parameters [42].

Table 2 illustrates the model fit for the various models. The IRTree model exhibited a lower AIC and BIC compared to the GRM, indicating a superior model fit of the IRTree model for item explanation. Upon identifying the response biases in the items, we evaluated their effects. Table 3 displays the correlations among the latent traits within the IRTree model. The correlation between the two response biases ($\:{\theta\:}_{M}$ and $\:{\theta\:}_{E}$), and the correlation between the agreements on team growth and fixed mindsets ($\:{\theta\:}_{ATG}$ and $\:{\theta\:}_{ATF}$) were both negative and moderate (− 0.567 and − 0.585), indicating the convergent validity of bias and agreements on each other [42]. On the other hand, the model had the weak correlations between response bias and team mindset agreements (− 0.316/0.325 for $\:{\theta\:}_{ATG}$ and 0.329/−0.316 for $\:{\theta\:}_{ATF}$), and it supported discriminant validity between the bias and agreement traits [42]. We explored items with inappropriate properties based on the IRTree model and response pattern. We deemed the items inadequate when the item parameters were insignificant. For example, a single-item TF mindset exhibited a minor parameter issue; we identified TFPERS4 as inappropriate in addition to the EFA result.

**Table 2:** Model fits of GRM and IRTree models

Model	Number of Parameters	AIC	BIC
GRM	241	43693.51	44632.06
IRTree	200	41550.18	42329.06

**Table 3:** Correlations of latent traits in IRTree models

	$\:{\boldsymbol{\theta\:}}_{\boldsymbol{A}\boldsymbol{T}\boldsymbol{G}}$	$\:{\boldsymbol{\theta\:}}_{\boldsymbol{A}\boldsymbol{T}\boldsymbol{F}}$	$\:{\boldsymbol{\theta\:}}_{\boldsymbol{M}}$	$\:{\boldsymbol{\theta\:}}_{\boldsymbol{E}}$
Agreement on Team Growth Mindset ($\:{\theta\:}_{ATG}$)	1			
Agreement on Team Fixed Mindset ($\:{\theta\:}_{ATF}$)	**−0.585** [−0.601, −0.569]	1		
Midpoint Response ($\:{\theta\:}_{M}$)	**−0.348** [−0.461, −0.235]	**0.329** [0.328, 0.329]	1	
Extreme Response ($\:{\theta\:}_{E}$)	**0.325** [0.207, 0.442]	**−0.316** [−0.413, −0.219]	**−0.567** [−0.627, −0.506]	1

All latent correlations were significant at *p* <.001; Values in parentheses indicate 95% confidence intervals; Bold values indicate statistically significant point estimates

**Fig. 3:**
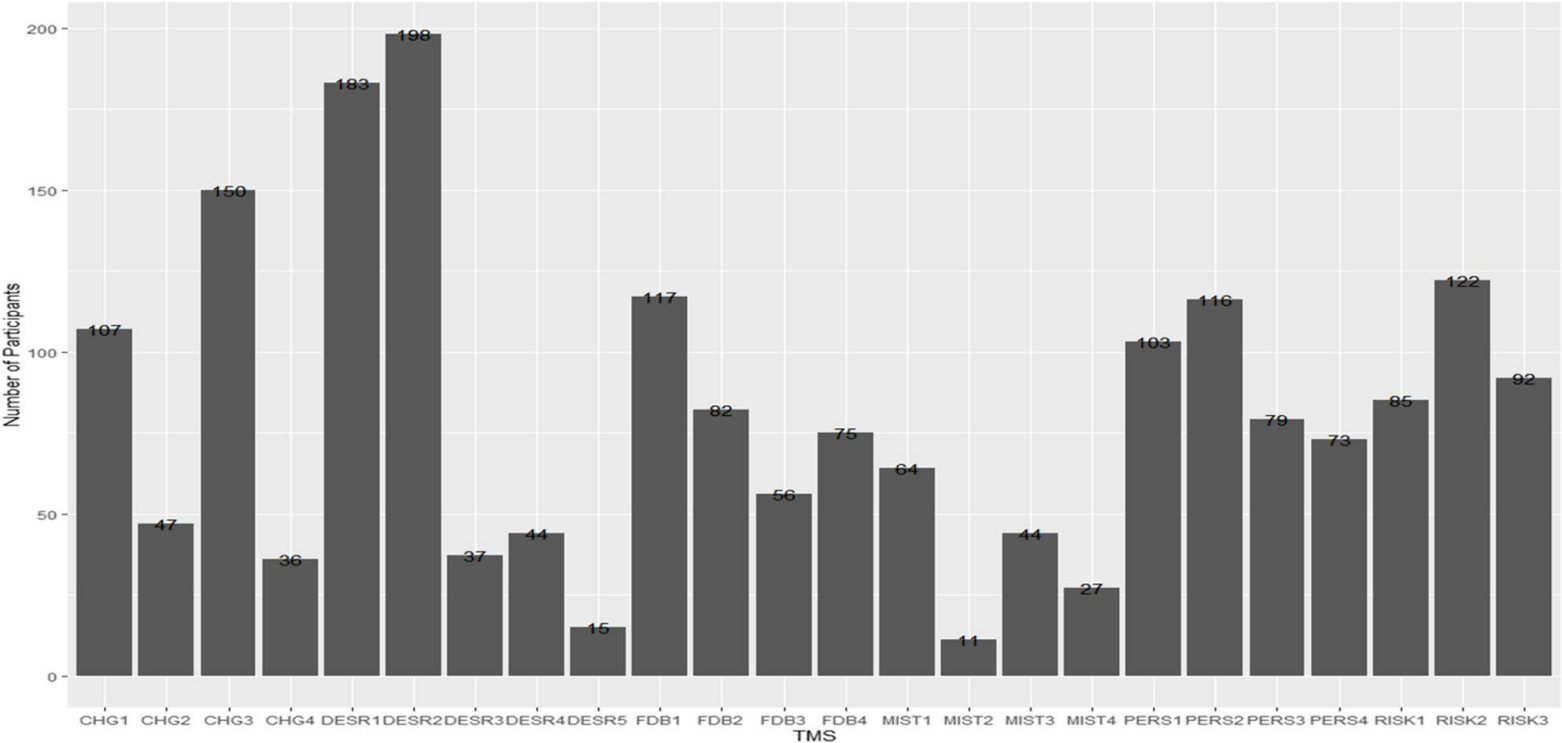
Frequencies of Agreement with Strongly Agree and Agree on TGM and TFM

We additionally compared agreement frequencies for the TGM and TFM items. We anticipated that the agreement responses to the TGM and TFM items within the same pair would differ due to their opposing meanings on the team mindset continuum. Agreement by the respondent on both TGM and TFM within the same pair signified item ambiguity. Figure 3 illustrates the frequencies of agreement for each item over the total number of participants. For instance, 198 participants responded *agree* or *strongly agree* to TGDESR2 and TFDESR2. TGM and TFM items were excluded if over 100 participants (28% of total responses) expressed agreement. The 16-item set (PERS1, PERS2, CHG1, CHG3, RISK2, FDB1, DESR1, and DER2) was excluded from the scale due to its inability to clearly differentiate among the two team mindsets.

Subsequent to the item screening process, the TGM and TFM retained nine items. We re-executed the EFA model utilizing these 18 items, corroborating a two-factor model. The two team mindsets exhibited a substantial negative factor correlation (− 0.49). Table 4 displays the factor loadings of the items based on the two-factor model.

**Table 4:** Factor loadings for team mindsets

	Team Growth Mindset		Team Fixed Mindset	
	Estimates	*p*-value	Estimates	*p*-value
TGPERS3	**0.439**	< 0.001	−0.133	0.251
TGCHG2	**0.527**	< 0.001	−0.055	0.674
TGRISK1	**0.641**	< 0.001	−0.146	0.287
TGFDB3	**0.626**	< 0.001	−0.068	0.629
TGFDB4	**0.715**	< 0.001	0.031	0.756
TGMIST1	**0.804**	< 0.001	0.078	0.607
TGMIST2	**0.541**	< 0.001	0.003	0.781
TGMIST4	**0.604**	< 0.001	0.06	0.649
TGDESR3	**0.702**	< 0.001	−0.07	0.628
TFCHG2	−0.157	0.139	**0.406**	< 0.001
TFCHG4	**−0.294**	0.002	**0.403**	< 0.001
TFRISK1	−0.168	0.136	**0.467**	< 0.001
TFFDB4	0.077	0.561	**0.627**	< 0.001
TFMIST1	−0.069	0.571	**0.407**	< 0.001
TFMIST3	−0.107	0.354	**0.334**	< 0.001
TFMIST4	−0.149	0.176	**0.371**	< 0.001
TFDESR3	0.010	0.750	**0.594**	< 0.001
TFDESR5	**−0.332**	< 0.001	**0.326**	0.001
**Factor Correlation**				
TG	1	-		
TF	**−0.494**	< 0.001	1	-

*TG* = Team Growth Mindset; *TF* = Team Fixed Mindset; *PERS* = Perseverance; *CHG* = Openness to Change; *RISK* = Taking Risk; *FDB* = Accepting Feedback; *MIST* = Learning from Mistakes; *DESR* = Desire to Learn from the Team; Bold indicates statistically significant point estimates

## Study 3: Testing the validities of the team mindset scale

According to Study 2, the 18 items constituted TMS for practical application. For cross-validation of the two-factor model, we assessed the construct validity of the TMS through confirmatory factor analysis (CFA). Furthermore, we conducted an examination of the measurement invariance of the TMS across genders. Finally, we examined TMS’s convergent and discriminant validities by comparing them with individual mindsets.

### Methods

The participants comprised undergraduate and graduate students from a public research university in the United States. The data collection procedure mirrored that of Study 2. Study 3 utilized 60% (*N* = 545) of the gathered data (*N* = 908, female = 61%, white = 82%).

### Measures

#### Team mindset

Study 3 examined the 18 team mindset items subsequent to the screening of the other items from Study 2. Cronbach’s alphas for the nine-item TGM and TFM were 0.89 and 0.81, respectively.

#### Individual mindset

The items were identical to those utilized in Study 1. In Study 3, Cronbach’s alphas for individual growth and fixed mindsets (i.e., IGM and IFM) were 0.94 and 0.90, respectively.

### Results

#### Confirmatory factor analysis

**Fig. 4:**
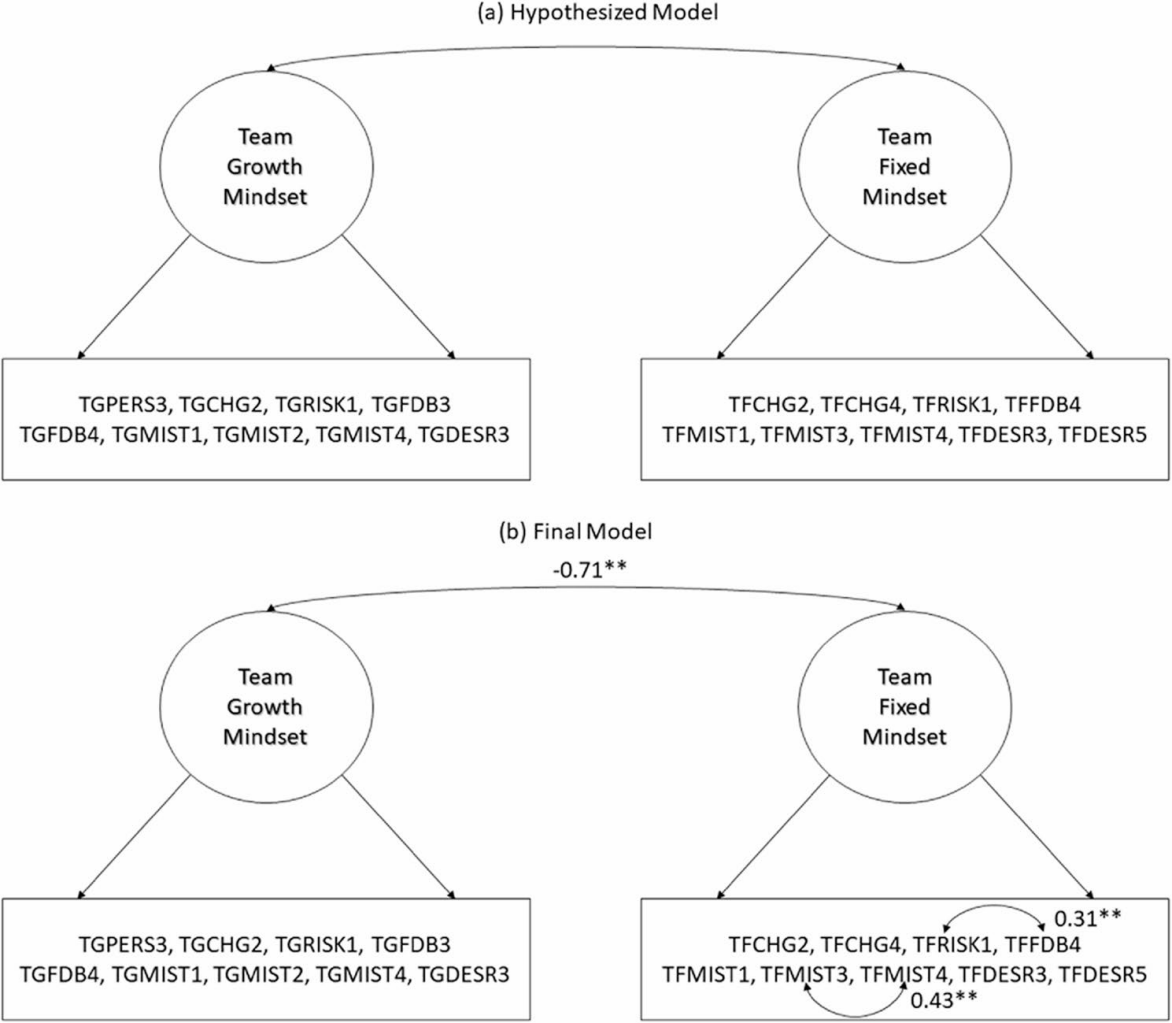
A Two-Factor CFA Model for the Team Mindset Scale

Study 3 employed CFA to validate the two-factor structure identified in Study 2. Figure 4 illustrates the test factor models. The model fit criteria (CFI > 0.95, RMSEA < 0.08, or SRMR < 0.05) [44] also corroborated the hypothesized two-factor model (a; Fig. 4) in Study 3 (CFI = 0.914, RMSEA = 0.060, and SRMR = 0.052; Table 5). However, we modified the model by sequentially releasing the correlation between unique factor variances to enhance model fit; consequently, we estimated the unique variance correlations between TFRISK1 and TFFDB4 (0.307, *p*-value < 0.001), and TFMIST3 and TFMIST4 (0.426, *p*-value < 0.001), resulting in a marginally improved model fit (CFI = 0.920, RMSEA = 0.064, and SRMR = 0.049). The correlations between TF3 and TF4, as well as TF6 and TF7 may stem from their analogous meanings. Each pair of items inquired about the dominant team member and the dominant solution.

**Table 5:** Model Fits of the Factor Analysis Models

Model		χ2_(df)_	*p* -value		CFI	RMSEA	SRMR
CFA for Team Mindset							
2-Factor Model		354.434 _(134)_	<.001		0.914	0.060	0.052
Modified 2-Factor Model		425.146 _(132)_	<.001		0.920	0.064	0.049
Measurement Invariance							
Gender							
Configural		Δχ2			0.915	0.066	0.055
Metric		16.328 _(16)_	0.430		0.915	0.064	0.060
Scalar		16.512 _(16)_	0.418		0.915	0.063	0.062
CFA for Team and Individual Mindset							
4-Factor Model		574.755 _(246)_	<.001		0.956	0.050	0.040

#### Measurement invariance

The measurement of team mindsets must remain consistent across all demographic subgroups. Measuring team mindsets uniformly across subgroups demonstrates measurement invariance, thereby reinforcing the validity of TMS [45, 46]. Based on the invariant parameters among subgroups, each phase of the measurement invariance model comprises configural, metric, and scalar invariance models [46]. Configural invariance indicates an identical factor analysis model devoid of any parameter constraints. Following the analysis that confirms configural invariance, the metric invariance model can be tested for equal factor loadings of items by constraining the factor loadings to be identical across groups. Besides factor loading invariance, the scalar invariance model examines the equality of item intercepts across groups. The mean differences of the items among groups are attributable to the factor mean difference solely if scalar invariance is maintained. Therefore, maintaining scalar invariance is essential evidence for the appropriate measurement of TMS.

We evaluated measurement invariance across genders utilizing the multi-group CFA model [46]. We examined each invariance model from configural to scalar, and conducted a χ^2^ difference test for model comparison. Table 6 presents the model fit for the assessment of measurement invariance in the testing measurement invariance. Δχ^2^ denotes the χ^2^ difference among the models (i.e., configure vs. metric and metric vs. scalar). All Δχ^2^ values were insignificant; the constrained parameters did not influence the model fit. The alternative model fits were also satisfactory; therefore, the scalar invariance of TMS is maintained across genders.

#### Convergent and discriminant validity

To examine whether we measured team mindsets distinctively from individual mindsets, we analyzed the four-factor model, which encompasses the two factors from the team mindset scale (TMS) and the individual mindset scale (IMS). The factor model was validated using an adequate model fit (CFI = 0.956, RMSEA = 0.050, SRMR = 0.040). Table 6 illustrates the correlations among the four factors. The factor correlations between team and individual mindsets were minimal (0.188/−0.174 between TGM and IGM/IFM, and − 0.191/0.249 between TFM and IGM/IFM). This finding corroborated the discriminant validity of TMS, indicating that team mindsets are distinct constructs from individual mindsets. In contrast, the growth and fixed mindsets within each team and individual mindsets exhibited significant negative correlations (− 0.769 between TGM and TFM; −0.823 between IGM and IFM). The negative sign denotes the contrary growth of the continuum and fixed mindset directions. In addition, the strong correlation among team mindsets bolstered the convergent validity of the TMS.

We refined the 18-item TMS based on various validities from Studies 2 and 3. Table 7 presents the conclusive 18-item TMS, encompassing the reliability and descriptive statistics of the TGM and TFM. Cronbach’s alphas for each team mindset were satisfactory (α > 0.70) across different samples from Study 2 and Study 3.

**Table 6:** Mindset factor correlations

Factor	TGM	TFM	IGM	IFM
Team Growth Mindset (TGM)	1	−	−	−
Team Fixed Mindset (TFM)	**−0.769** [−0.821, −0.718]	1	−	−
Individual Growth Mindset (IGM)	**0.188** [0.103, 0.278]	**−0.191** [−0.282, −0.095]	1	-
Individual Fixed Mindset (IFM)	**−0.174** [−0.264, −0.085]	**0.249** [0.156, 0.342]	**−0.823** [−0.856, −0.789]	1

All *p*-values were < 0.001; Values in parentheses indicate 95% confidence intervals; Bold indicates statistically significant point estimates

**Table 7:** Final items of the team mindset scale

Item	Statement	Reliability of α (Item means and standard deviation)
TGM1 (TGPER3)	Our team created milestones and celebrated small successes.	Study 2 α = 0.86 (*M* = 3.66, *SD* = 1.03) Study 3 α = 0.89 (*M* = 3.60, *SD* = 1.08)
TGM2 (TGCHG2)	Our team combined ideas from multiple sources to make a unique project.	
TGM3 (TGRISK1)	Our team managed challenges through a joint effort.	
TGM4 (TGFDB3)	Our team considered alternative solutions.	
TGM5 (TGFDB4)	Our team resolved conflicting ideas by hearing multiple arguments.	
TGM6 (TGMIST1)	Our team actively learned from obstacles.	
TGM7 (TGMIST2)	Our team valued failure as an opportunity to learn.	
TGM8 (TGMIST4)	Our team diagnosed the causes when we encountered a major problem.	
TGM9 (TGDESR3)	Our team had many different perspectives that added to the entire process.	
TFM1 (TFCHG2)	Our team felt that contrary opinions inhibited our efficiency.	Study 2 α = 0.77 (*M* = 2.67, *SD* = 1.11) Study 3 α = 0.81 (*M* = 2.69, *SD* = 1.16)
TFM2 (TFCHG4)	Our team avoided debating ideas.	
TFM3 (TFRISK1)	Our team relied on a key individual to solve challenges.	
TFM4 (TFFDB4)	Our team resolved conflict through solutions from dominant members.	
TFM5 (TFMIST1)	Our team experienced similar problems multiple times.	
TFM6 (TFMIST3)	Our team held on to the same solution idea throughout the entire process.	
TFM7 (TFMIST4)	Our team stayed with the first model we developed despite it having problems.	
TFM8 (TFDESR3)	Our team experienced inefficiencies in our processes because each member exhibited a different perspective.	
TFM9 (TFDESR5)	Our team did not listen to my opinion.	

## General discussion and future directions

We presented the findings of three studies aimed at developing a scale to measure team mindsets. First, we aimed to develop each set of items for team growth and fixed mindsets; therefore, we selected final items that exhibited suitable characteristics and validities from the initial items. Subsequently, to validate the new scale, we conducted three independent studies utilizing distinct samples from workplace and higher education settings. Finally, we recommended item sets for the TMS subscales derived from the results. Analysis of convergent and discriminant validity demonstrated that the factor correlations among the TMS subscales exceeded those between IMS and TMS. Therefore, this result validated the convergent and discriminant validities of the TMS.

Specifically, the EFA in Study 1 revealed that team and individual mindsets may possess distinct factor structures, indicating that individuals perceive individual mindsets differently from team mindsets. Further analysis was required to establish the optimal team mindset items that meet the requisite psychometric properties. As Study 1’s sample was too small for assessing item properties, we gathered responses from a different cohort of participants to access a larger sample and analyzed the data in Studies 2 and 3. In Study 2, we assessed whether the final set of items possessed adequate item characteristics. We selected items based on the psychometric properties from the EFA and IRTree models, as well as content validity. In Study 3, we investigated various forms of the final scale’s validity CFA. In pursuit of a better fit of the TMS measurement model, we identified significant factor correlations among TFM3 and TFM4, as well as TFM6 and TFM7. Researchers may either selectively utilize all four items or exclude one of the correlated items. We detected no measurement bias in TMS across genders through the evaluation of measurement invariance. In addition, convergent and discriminant validity assessments confirmed a clear distinction between the TMS and the IMS.

### Theoretical contributions

This study aimed to make a theoretical contribution by identifying the mindsets individuals should adopt to cultivate empowered teams. We commenced with an individual mindset and subsequently expanded the scale to encompass team mindsets. The results demonstrated that team mindsets are quantifiable constructs distinct from the aggregated data of IMS. Factor analyses in our studies adequately supported the two-dimensional constructs of team mindsets (TGM and TFM). Further, we obtained the cross-validation of two independent samples. The three studies affirm the generalizability of the instrument to a wider array of organizations and workplaces.

Considering the significance of teams in work and educational environments, exploring the connection between individual and team mindsets, as well as analyzing the dimensions of team mindsets, would yield valuable insights for practical application in higher education. Our developed TMS can expand future research to explore the relationships between individual mindsets, team mindsets, and other essential variables, including team communication and workplace creativity [47]. In addition, researchers can broaden the analysis to encompass various adult learning and organizational contexts.

### Practical implications

The team mindset scale serves as a diagnostic tool for practitioners to identify characteristics of a growth mindset within teams. Understanding one’s mindset may enhance organizational team performance or work. Practitioners can utilize this scale to assist employees or members engaged in teamwork in developing awareness of their mindsets, whether a team growth mindset or a team fixed mindset. Awareness of mindsets can enhance the work environment, encourage employees to foster a growth mindset within teams, and potentially contribute to improved organizational culture and performance [48].

### Limitations and future research

We acknowledge the limitations of this study and recommend avenues for future research. First, researchers can expand the sample size and diversity. Moreover, subsequent research should test the generalizability of a TGM using samples derived from diverse rating sources and organizations beyond those that we incorporated (i.e., governmental work place and higher education). Given that this is a team mindset scale, incorporating diverse perspectives from subordinates and supervisors may prove beneficial. Incorporating self-ratings and peer/team ratings may enhance the precision of the scale.

Further, we developed TMS to measure individuals’ perceptions of their team’s mindset. The team mindset constitutes a distinct construct from that of an individual’s mindset. Therefore, investigating the similarities and differences among the two constructs is essential. However, due to the limited team size (i.e., team members for each team), this study concentrated on individual-level data analysis. Future studies utilizing team-level analysis could yield additional insights into the distinctions between team mindsets and individual mindsets, as well as their relationships with other team-level constructs. In addition, we recommend incorporating social desirability items in subsequent surveys to mitigate errors and enhance the validation process.

## Data Availability

The datasets are available in OSF (https://osf.io/zr24m/?view_only=e322c9327bee40259ca93638e1f8ecde).
